# Professional identity and job performance among vocational education teachers—a chain mediation model of leader–member exchange and self-efficacy, with job crafting as a moderator

**DOI:** 10.3389/fpsyg.2026.1758017

**Published:** 2026-06-05

**Authors:** Ying Zhou, Zonglin Zhong

**Affiliations:** Office of Discipline Development, Jiangxi Institute of Technology, Nanchang, China

**Keywords:** job crafting, job performance, leader–member exchange, professional identity, self-efficacy

## Abstract

**Purpose:**

This research explores the effect of professional identity (PI) on the job performance (JP) of vocational education and training (VET) teachers, with a particular focus on the sequential mediation effects of leader-member exchange (LMX) and self-efficacy (SE). It also explores the role of job crafting (JC) as a moderator of these sequential mediation effects.

**Method:**

A questionnaire using a Likert scale was designed and distributed to 2,392 teachers from six vocational colleges in China. Correlation analysis and moderated mediation analysis were performed using SPSS 20.0 and AMOS 22.0 to test the hypotheses.

**Results:**

PI, SE, LMX and JP were significantly correlated (*p* < 0.05). PI had a significant positive impact on JP. Further, LMX and SE were sequential mediators between PI and JP, with SE having a greater impact on the indirect effect than LMX. Finally, JC significantly moderated the sequential mediation effect (PI → LMX → SE → JP); however, it did not significantly moderate the simple mediation effect (PI → LMX → JP).

**Conclusion:**

PI has a direct and indirect effect on the JP of VET teachers. The indirect effect is mediated by a sequential mediation pathway (LMX and SE). Moreover, JC serves as a boundary condition that moderates specific pathways of the sequential mediation process (PI → LMX → SE → JP), demonstrating a pathway-specific and constraining moderating effect.

## 1 Introduction

Vocational education is a vital means of providing the skilled workforce needed for industrial and economic growth (Hofmeister and Pilz, 2020; Reimers, 2022). In a rapidly evolving technological environment, and with the growing need for a range of skills in the labor market, vocational education has become increasingly important (Kovalchuk et al., 2022). In this regard, teacher job performance (JP) is acknowledged as a critical factor in education quality (Nugroho et al., 2023). Yet, vocational education teachers are currently facing multiple challenges, such as a lack of institutional resources, high job demands and limited career development opportunities (Adam and Mengistie, 2026; Rice et al., 2025). As a result, they increasingly need to draw on their psychological resources and proactive strategies to maintain their performance. This highlights the need to explore the role of various types of resources in JP in these contexts.

Among these factors, professional identity (PI) has been identified as a key predictor of teacher performance (Vähäsantanen and Hämäläinen, 2019; Yu et al., 2025). Existing studies indicate that a strong sense of PI increases teachers' work commitment, facilitates effective teaching practices, and boosts motivation to meet teaching and learning goals (Suarez and McGrath, 2022). Furthermore, some researchers contend that PI fundamentally alters teachers' attitudes and behaviors, serving as both a pedagogical and developmental mindset that ultimately impacts student learning outcomes (Rosdi et al., 2020; Zhai et al., 2024). Crucially, a strong PI also acts as a psychological buffer, allowing teachers to cope with work stress and sustain their performance by conserving emotional resources (Xing, 2022). While there is general agreement about the importance of PI, the theoretical pathways by which PI leads to sustained performance outcomes are not well understood.

To bridge this gap, researchers have highlighted the critical function of social and psychological resources in shaping individuals' JP (Ochoa Pacheco et al., 2023; Yen et al., 2020). Viewed through a relational perspective, leader–member exchange (LMX) captures the quality of interaction and trust between leaders and followers, and researchers have shown links between high-quality exchange relationships and a variety of positive outcomes at work, such as job satisfaction, engagement and performance (Khiong, 2023; Supriyanto et al., 2021). Psychologically, researchers largely regard self-efficacy (SE) and individuals' beliefs in their capacity for accomplishing work tasks, as an important antecedent of JP (Akman, 2021). Concurrently, research suggests that employees can improve their performance by proactively reshaping their work tasks, interpersonal relationships, and perceptions of work (Moreira et al., 2022). While prior empirical work has documented the individual impacts of these factors in relation to teachers' JP, they have been treated as separate influences. Consequently, existing research lacks a comprehensive explanatory model detailing how PI shapes JP through the combined operation of these mechanisms.

Adopting a resource-based perspective, this paper utilizes Conservation of Resources (COR) theory as its guiding framework and analyzes the multi-stage generation and transformation of resources through complementary theoretical perspective. Specifically, this paper conceptualizes PI as a core motivational resource resource that influences both individual attitudes and JP, fosters the development of relational resources (e.g., LMX) and psychological resources (e.g., SE), and facilitates resource acquisition and transformation at various levels.

When job resources are scarce, vocational education teachers tend to rely more on internal psychological resources and self-motivated behaviors to sustain their JP. Therefore, this study introduces job crafting (JC) as a key contextual moderator in the mediation model.

In the context of vocational education teachers, this study develops a framework that integrates the sequential relationships among relational resources, psychological resources, and performance outcomes, thereby specifying the roles and interrelationships of each mechanism within the overall process. Beyond advancing our comprehension of the mechanisms linking PI to JP, this study offers a more comprehensive theoretical framework to conceptualize how teachers' performance is formed through resource transformation.

## 2 Literature review and hypotheses

### 2.1 Professional identity

The PI of VET teachers is conceptualized as a multifaceted and dynamic construct. It encompasses the ongoing awareness and interpretation of a teacher's professional role and value, forged through the interaction between self-perception and external influences over the course of a career (Solari and Martín Ortega, 2022), and reflects deeply internalized personal understandings of professional goals, values, beliefs, and emotions (Yakov et al., 2021). Previous researchers has identified four fundamental attributes of identity: its socially constructed nature, its continual revisability, its diversity, and its embeddedness in the interpretation and description of experience (Luehmann, 2007).

A growing number of literature demonstrate that teacher identity is pivotal in shaping a teacher's professional development, career dedication, and instructional quality (Huang and Wang, 2024; Wallin et al., 2022). Teachers with higher levels of PI are more likely to display higher levels of pedagogical enthusiasm, job satisfaction, and lower levels of burnout and turnover intentions (Alon and Bergman Deitcher, 2024; Falcón Linares and Arraiz Pérez, 2020; Zhang et al., 2022). Moreover, empirical research suggests that PI has a direct impact on teachers' teaching practices and performance (Abednia, 2012; Lutovac, 2020). Crucially, PI provides the intrinsic motivation needed for teachers to view their profession as a calling. It allows them to adapt more readily and successfully to the ever-changing educational landscape and to engage more creatively and proactively in educational reform (Richter et al., 2021; Simon, 2024; Su, 2023).

### 2.2 PI and JP

JP is the collective manifestation of behaviors and outcomes that employees use to achieve work-related objectives (Bentley et al., 2017). Given that PI reflects individuals' self-definition in their occupational group, it is a critical factor in shaping their values and norms, which in turn has a significant impact on JP (Zhao, 2022). Social Identity Theory suggests that group membership forms the foundation for self-identity. When individuals identify strongly with a professional group, they strive to uphold a positive group image by showing in-group favoritism and intergroup comparison. In turn, these processes lead to increased work engagement and JP (Trepte and Loy, 2017). In particular, teachers with a well-developed PI are more likely to follow professional norms and engage in discretionary effort to improve the status and reputation of the teaching profession. These actions, in turn, lead to better individual performance and, ultimately, to better educational outcomes (Ellemers and Rink, 2005). Research also suggests that teachers with a strong PI are more likely to feel passionate about their work, be more committed to student learning, and better understand and perform their professional roles, leading to higher JP (Wu et al., 2024).

In terms of Self-Determination Theory, intrinsic motivation is a critical factor underlying engagement-related actions, and PI serves as a core internal resource that activates intrinsic motivation. In turn, increased intrinsic motivation leads to higher job satisfaction (Van Der Wal et al., 2019), work engagement (Butakor et al., 2021), and innovation (Beijaard et al., 2004). Existing evidence has shown that job satisfaction, work engagement and innovation are important factors in predicting teachers' JP (Balbes and Quines, 2022; Vuong et al., 2023). Drawing on these theoretical insights and empirical evidence, we put forward the following hypothesis:

H1: PI has a positive effect on VET teachers' JP.

### 2.3 The mediating role of LMX

Leader-member exchange (LMX) is the interpersonal relationship between leaders and subordinates, formed through a series of reciprocal interactions. Over time, the leader and the subordinate mutually influence each other, co-constructing the subordinate's role and status in the organization (Jufrizen et al., 2024). LMX has emerged as a key construct in organizational behavior, capturing variation in the quality of leader-follower interactions (Graen and Uhl-Bien, 1995). According to Social Identity Theory, high role and organizational identification leads to a greater sense of belonging and a greater awareness of occupational norms and values. This leads to increased collaboration and more frequent and high-quality interpersonal interactions, improving workplace relationships (Spears, 2021). Research also shows that PI is strongly related to interpersonal workplace relationships, with LMX being a critical interpersonal construct (Wu et al., 2025).

Workers with high PI also exhibit greater task performance, which promotes more frequent and high-quality interactions with their leaders (Martin et al., 2023). These interactions are crucial to the formation of high-quality LMX relationships (Dulebohn et al., 2012). LMX theory also suggests that variations in interaction quality result in the creation of in-groups and out-groups (Martin et al., 2018). In-group members tend to enjoy more trust, support and resource allocation than out-group members (Omilion-Hodges and Ptacek, 2021). These differences in relationships have important consequences for JP, job satisfaction, career advancement, and organizational loyalty. Additionally, employees with high-quality LMX relationships are often afforded more freedom and job resources, which further boosts their performance (Jufrizen et al., 2024). Theoretically, LMX is a mutually beneficial relationship, and its quality is often assessed along four dimensions: affect, contribution, loyalty, and professional respect (Liden and Maslyn, 1998).

According to Social Exchange Theory, social exchanges are reciprocal and mutually beneficial (Ahmad et al., 2023). Thus, when VET teachers are engaged and persistent in their work, leaders are more likely to view them as trustworthy and valuable, and thus provide greater trust, support and resources, which in turn increases teachers' JP (Lin et al., 2024). Indeed, a large body of empirical evidence supports the positive relationship between LMX and JP (Ionescu and Iliescu, 2021; Martin et al., 2016). For example, in a multi-wave study of undergraduate and graduate social work students, Thrasher et al. (2020) found LMX moderated the effects of interpersonal exchanges on supervisor-rated JP, which in turn impacted performance. Drawing on these theoretical insights and empirical evidence, LMX is anticipated to serve as a key relational factor between PI and JP. As such, the following hypothesis is put forward:

H2: LMX mediates the relationship between VET teachers' PI and JP.

### 2.4 The mediating role of SE

SE is defined as a person's fundamental belief in their ability to successfully complete a task (Woodcock and Tournaki, 2023). As outlined above, teachers with a high sense of PI are more likely to be optimistic and resilient in the face of teaching challenges (Namaziandost et al., 2022). These teachers are more likely to engage in proactive and creative teaching strategies, which further enhances their efficacy beliefs (Marschall, 2022). Furthermore, teachers with a strong PI are more likely to engage with leaders and peers, creating a network of support. They seek out resources, support and encouragement from these networks, which are essential for the development of SE (Polizzi et al., 2021). This is supported by the literature. For example, Van Der Want et al. (2019) reported that interpersonal role identity is positively related to teachers' SE and engagement, and negatively related to burnout.

Following on from this, VET teachers with a greater PI are more likely to display higher confidence in their teaching roles. Moreover, previous studies suggest that people with high SE are more likely to engage in more positive cognitive appraisals in response to challenges, which increases their ability to cope and the likelihood of better JP (Bernales-Turpo et al., 2022). Teachers with high SE are confident in their teaching, classroom management, and ability to promote student learning. Crucially, they tend to view educational reforms as opportunities for growth. In the face of pedagogical or research-related challenges, they are more likely to seek out learning opportunities, try out new teaching strategies, and are more flexible and adaptable, which are all linked to high JP (Gordon et al., 2023).

Beyond its impact on teaching practices, SE is also a key resource for emotional regulation and stress coping. Teachers with higher SE are better equipped to regulate their emotions and cope with stressors in the workplace (Li, 2023). As a result, they are more likely to adopt a proactive and problem-focused approach to professional challenges, rather than avoidant or withdrawal strategies (Waddington, 2023). These positive dispositions and behaviors are essential for improving professional performance.

Drawing upon this mechanistic rationale, we formulate the following hypothesis:

H3: SE mediates the relationship between PI and JP.

### 2.5 The chain-mediating role of LMX and SE

Drawing on Social Exchange Theory (Zhang and Wang, 2021), people with high PI are more likely to put in the effort to build strong relationships with their colleagues (Wu and Lv, 2024). These relationships offer crucial interpersonal resources, including feedback, support and recognition, which in turn boost teachers' self-efficacy (Martin et al., 2016; Zheng et al., 2023). In turn, higher SE motivates teachers to invest more discretionary effort and sustain their work efforts, which leads to higher JP (de Oliveira Fernandez et al., 2016).

According to the Job Demands-Resources (JD-R) theory, people are naturally motivated to gain, maintain and protect valued job resources. In this model, the presence of adequate resources in the workplace is essential in motivating individuals and improving work performance (Bakker and Demerouti, 2017).

VET teachers may face unique structural challenges compared to their peers in general education, such as resource scarcity, job insecurity and limited career advancement opportunities (Nakar and Du Plessis, 2023). In this context, the resources of recognition, information and development opportunities offered by organizational leaders are particularly important for career advancement (Collie and Martin, 2017). High-quality LMX relationships represent a key avenue for accessing these resources, such as time, support and information, which allow teachers to better tackle professional challenges (Fang et al., 2025). These resource gains not only boost teachers' SE but also improve their teaching performance and innovation. By bolstering teachers' belief in their abilities, high-quality LMX relationships support ongoing performance enhancement and more flexible adaptation to the challenges of vocational teaching.

Based on the above theoretical arguments and empirical evidence, the following hypothesis is proposed:

H4: LMX and SE sequentially mediate the relationship between PI and JP.

### 2.6 The moderating role of JC

JC is defined as employees' proactive changes to their job tasks, interpersonal interactions, and job-related thoughts to improve person-job fit (Wrzesniewski and Dutton, 2001). It serves as a means to activate more resources needed to complete tasks and to decrease job demands. JC has been proven to be an effective proactive resource-building strategy in previous studies (Tims et al., 2022). COR theory suggests that people are naturally inclined to obtain, retain, protect and build resources (Niazi et al., 2024). Consequently, teachers with high PI are more likely to proactively engage in JC by adjusting their role perceptions, work tasks and interactions. In doing so, they can mobilize latent structural and relational resources, such as time, material and social resources, to complete their work more efficiently (Zhai et al., 2025). This resource maximization serves as a buffering effect that helps to reduce work-related difficulties and constraints, leading to better JP (Bakker et al., 2023). Additionally, empirical research suggests that JC increases the fit between job demands and individual needs, resulting in greater job satisfaction, PI, and better performance and behavioral outcomes among teachers (Holman et al., 2024).

Drawing on these theoretical and empirical findings, this study suggests that JC is a moderating factor that enhances the positive association between PI and JP among VET teachers. Thus, we propose the following hypothesis:

H5: JC moderates the relationship between PI and JP.

Moreover, research indicates that people who engage in adaptive efforts, such as actively modifying work processes or improving workplace interactions, are more likely to attain individual and organizational goals. Such adaptive actions also boost the chances of gaining leaders' recognition and support, which in turn improves LMX relationships (Geldenhuys et al., 2021).

While some studies suggest that JC has a direct effect on LMX, other studies suggest that adaptive efforts such as changing job tasks, setting new goals and enhancing work relationships primarily enhance teachers' beliefs in their job-related abilities (Yang et al., 2021). This, in turn, enhances teachers' PI and JP (Mokhtar et al., 2023). Additionally, previous research shows that JC not only increases teachers' job satisfaction and PI but also increases their work engagement and JP (Holman et al., 2024). Overall, these studies highlight the critical role of JC in linking teachers' intrinsic motivation to their job performance.

Based on the synthesized theoretical and empirical evidence, we hypothesize that in the present study, JC will play a pivotal moderating role within the sequential mediation model linking VET teachers' PI, LMX and SE. Accordingly, the following hypothesis is proposed:

H6: JC moderates the chain mediation of LMX and SE in the relationship between PI and JP.

**Figure 1:**
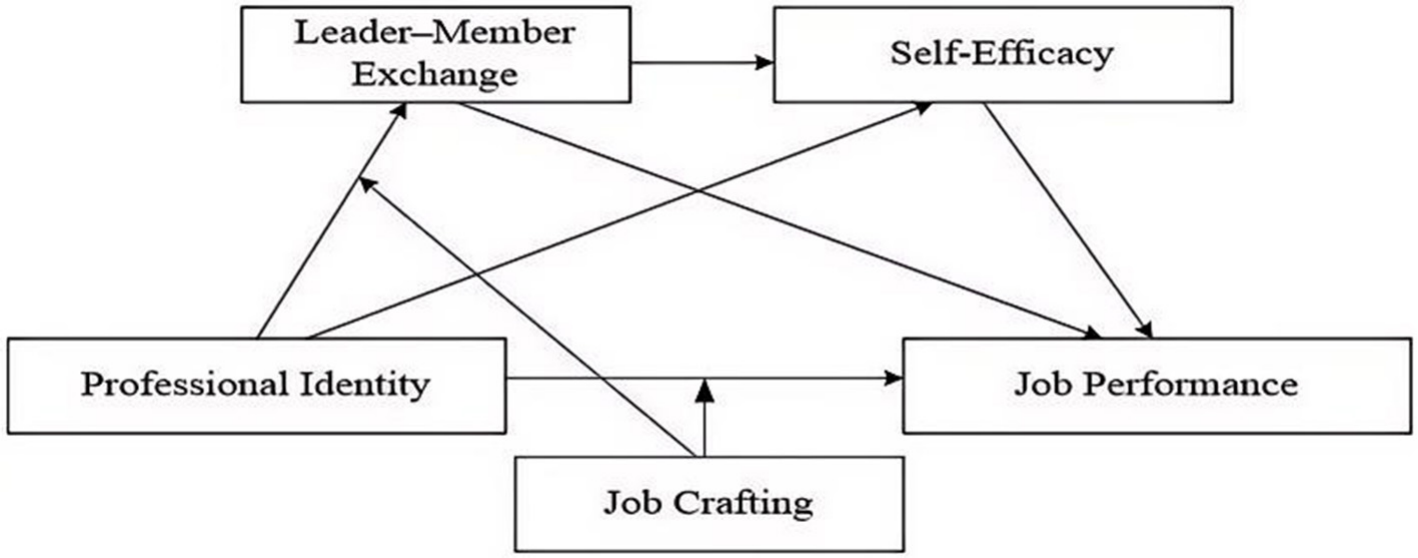
Measurement model. Factor loadings are standardized results (2,392). LME, leader–member exchange; SE, self-efficacy; PI, professional identity; JC, job crafting; JP, job performance.

In summary, although numerous studies have examined the isolated relationships among PI, LMX, SE, JC, and teachers' JP, no study to date has integrated these constructs to examine their interplay within a single framework. Building on prior findings, this study hypothesizes that PI influences both LMX and SE, which in turn affect the JP of VET teachers, with JC serving as a critical moderating boundary condition within this conceptual framework. Therefore, this study proposes the development of a conceptual model (see Figure 1) to empirically examine the complex interdependencies among these five variables.

## 3 Methodology

### 3.1 Research design

To systematically investigate the association between PI and JP, the mediating roles of LMX and SE, and the moderating function of JC, the following research design was adopted. First, a grounded approach was employed to explore the relationship between PI and JP. Concurrently, interviews were conducted to explore the potential mediating effects of LMX and SE, along with the possible moderating influence of JC, while an exhaustive review of relevant literature was conducted to derive the corresponding hypotheses. Second, the minimum required sample size was estimated based on teachers' disciplinary diversity and the total number of questionnaire items, prior to determiningan appropriate sample size through stratified random sampling. Furthermore, the questionnaire was submitted to the institutional ethics committee for ethical clearance. Third, following the standard procedures for questionnaire administration, we explained the purpose of the study to participants; afterwards, participants were asked to fill in and sign an informed consent form, retaining the right to withdraw from the study at any time. Finally, the data verification and model analysis were executed.

### 3.2 Participants and procedure

The data collection method for this study was online and paper-based questionnaires. To improve the measurement quality, the data were re-collected in March 2026 from vocational education teachers with more than 1 year of teaching experience in Jiangxi, Chongqing and other areas. The variables were measured in a single wave, which enhances internal validity and reduces the risk of common method bias (CMB).

We sent out 2,500 questionnaires. Following the removal of invalid or incomplete questionnaires, 2,392 valid questionnaires were left, for an effective response rate of 95.7%.

The sample's descriptive statistics were as follows. By gender, 33.7% were men and 66.3% were women. Regarding age distribution, 9.9% were under 25 years old, 36.3% were between 26 and 30, 29.6% were between 31 and 40, 18.7% were between 41 and 50, and 5.5% were over 50 years old. In terms of marital status, 58.1% were married and 41.9% were single. In terms of academic rank, 2.7% were professors, 14.5% associate professors, 25.2% lecturers, and 27.1% assistant lecturers, while 30.6% had no academic titles. These ratios suggest that the sample is representative of VET teachers in China, as it captures the major demographic and professional features of this group.

### 3.3 Measures

All construct measures employed in this study were derived from validated instruments commonly used in earlier research, ensuring robust reliability and validity. Slight lexical adaptations were made to contextually align the scales with the VET context. For instance, the term “organization” was replaced with “vocational institution,” and items measuring “JP” were contextualized to reflect teachers' specific work responsibilities (e.g., teaching, research, and related professional activities). Except where explicitly stated, all items were retained without addition or deletion. To further validate the appropriateness of the adapted measures, a confirmatory factor analysis (CFA) was performed, yielding results that supported the preservation of their original structural properties. Responses were recorded using a five-point Likert scale, with values ranging from 1 (strongly disagree) to 5 (strongly agree), unless stated otherwise. A complete inventory of measurement items is available in the Supplementary material.

This study employed PI scale developed by Wang (2022) to measure PI among vocational education teachers. With its clear structure, well-defined constructs, and concise design, the scale effectively avoids item redundancy. Furthermore, by focusing on the core dimensions of PI, it enhances the measurement accuracy within the context of vocational education. In addition, minor adjustments were made to improve its contextual appropriateness. The scale demonstrated a Cronbach's α = 0.969. Confirmatory factor analysis (CFA) and internal consistency testing, the scale demonstrated excellent reliability (0.973) and robust construct validity. The fit indices are as follows: χ^2^*/df* = 3.08, *CFI* = 0.997, *TLI* = 0.995, *NFI* = 0.996, *RMSEA* = 0.029, *RMR* = 0.012.

The Teacher JP Measurement Scale was adapted from the instrument developed by Cao (2018), originally designed for university faculty members, and was contextually refined to reflect the unique work context of VET teachers. The final adapted version included 12 items. The scale demonstrated a Cronbach's α = 0.964. Scale reliability and construct validity were rigorously established through confirmatory factor analysis (CFA) and internal consistency analysis of the scale. Corroborating the high reliability (0.964), the construct validity of the scale was indicated as follows: χ^2^*/df* = 6.554, *CFI* = 0.986, *TLI* = 0.980, *NFI* = 0.983, *RMR* = 0.040, *RMSEA* = 0.048.

The SE Scale applied in this study was derived from the Chinese edition of the General SE Scale developed by Wang (2002), incorporating context-specific modifications for VET teachers. The revised scale consisted of nine items. The scale demonstrated a Cronbach's α = 0.954. Following confirmatory factor analysis (CFA) and internal consistency testing, the scale demonstrated excellent reliability (0.954) and adequate construct validity. The fit indices were as follows: χ^2^*/df* = 5.08, *CFI* = 0.998, *TLI* = 0.993, *IFI* = 0.998, *GFI* = 0.995, and *RMSEA* = 0.041.

LMX was assessed using a revised form of the LMX-7 scale originally developed by Graen and Uhl-Bien (Hanasono, 2017), incorporating minor cultural and linguistic adaptations to suit the Chinese VET context. The finalized scale comprised eight items. The scale demonstrated a Cronbach's α = 0.928. The measurement model was evaluated through confirmatory factor analysis (CFA) and internal consistency testing, yielding satisfactory reliability (0.928) and robust construct validity. The fit indices are as follows: χ^2^*/df* = 13.09, *CFI* = 0.992, *TLI* = 0.982, *NFI* = 0.992, *RMSEA* = 0.071, and *RMR* = 0.013.

JC was assessed using a questionnaire derived from the scale developed by Slemp and Vella-Brodrick (2013). A total of 15 items were selected and adapted to reflect the specific work characteristics of VET teachers. The scale demonstrated a Cronbach's α=0.928. Following confirmatory factor analysis (CFA) and internal consistency testing, the scale demonstrated excellent reliability (0.968) and construct validity. The fit indices emerged as follows: χ^2^*/df* = 4.90, *CFI* = 0.995, *TLI* = 0.990, I*FI* = 0.995, *GFI* = 0.987, and *RMSEA* = 0.040.

The χ^2^/df values for certain measurement models exceeded conventionally recommended thresholds; however, this index is highly dependent on sample size, and χ^2^ values is notoriously sensitive to large sample sizes (Hu and Bentler, 1999; Kline, 2023). Therefore, greater emphasis was given to incremental and absolute Fit indices in evaluating model fit. The CFI, TLI, and RMSEA all fell within acceptable ranges, indicating an acceptable overall model fit.

### 3.4 Control variables

Following previous studies, this study controlled for a number of demographic factors to reduce their potential influence on the relationships between the key variables (Becker, 2005; Bernerth and Aguinis, 2016). In particular, age is related to work experience and may affect PI and JP. Gender may influence interpersonal interactions and LMX. Education is related to professional skills and SE, which influence JP. Moreover, marital status and annual income can be seen as measures of individual stability and life satisfaction, which are related to psychological resources (such as SE) and work performance. Academic rank is an indicator of career development and status within the organization and is also associated with differences in PI and JP.

As such, controlling for these variables helps to minimize the effects of potential confounding variables and improve the validity of the estimated relationships in this study.

### 3.5 Data analysis

The reliability and validity of the instruments were evaluated using SPSS 25.0 and AMOS 22.0. The findings showed that the standardized factor loadings for PI, JP, SE, LMX, and JC among vocational education teachers were all greater than 0.50, suggesting that the items were well represented by the constructs. The average variance extracted (AVE) for all constructs ranged from 0.601 to 0.677, and composite reliability (CR) ranged from 0.902 to 0.931. These values were higher than the recommended cut-off values (Fornell and Larcker, 1981), suggesting acceptable internal consistency and convergent validity.

Additional tests showed that the square roots of the AVE for each construct were greater than the inter-construct correlation coefficients, thus demonstrating satisfactory discriminant validity. While some of the correlation coefficients were relatively high, this is theoretically expected as the constructs are closely related but conceptually different, which is typical in organizational behavior studies. Given the relatively high CR values for some of the constructs, further analyses were performed to examine the possibility of item redundancy and to ensure measurement accuracy. The findings indicated that the average inter-item correlations were acceptable and only a few item pairs had very high correlations (*r* >0.85). Thus, the high CR values reflect high internal consistency rather than redundancy among items.

To determine whether there was any systematic bias arising from the methodology, the study conducted a Harman one-factor analysis on the data. The results showed that 55.6% of the variance could be explained by a single factor. Suggesting that common method variance might be a potential concern. Given the limitation of this technique (Howard et al., 2024; Podsakoff et al., 2003), confirmatory factor analysis was further employed to test the data. The results of the confirmatory factor analysis for the competing measurement models are presented in Table 1. The CFA results demonstrated that the five-factor model yielded superior fit indices, which were χ^2^*/df* = 4.331, *RMSEA* = 0.037, *CFI* = 0.968, *GFI* = 0.959, and *NFI* = 0.936. The fit indices of the competing models were substantially inferior, further corroborating the high construct validity and discriminant validity of the model.

**Table 1:** Results of confirmatory factor analysis for competing measurement models.

Model	Factor	x2/df	RMSEA	CFI	NFI
Single-factor model	A+B+C+D+E	9.402	0.059	0.92	0.911
Two-factor model	A+B+C+D, E	8.083	0.054	0.933	0.924
Three-factor model	A+B+C, D, E	7.301	0.051	0.94	0.931
Four-factor model	A+B, C, D, E	6.253	0.047	0.95	0.941
Five-factor model	A, B, C, D, E	4.331	0.037	0.968	0.959

A represents job performance; B represents job restructuring; C represents vocational identity; D represents leader-subordinate exchange; E represents self-efficacy.

We used SPSS 25.0 and AMOS 22.0 for data analysis. First, descriptive statistics and Pearson correlation analyses were performed to understand the means, standard deviations, and correlations between the variables. Second, confirmatory factor analysis (CFA) was conducted using AMOS 22.0 to evaluate the measurement model's reliability and validity. Once the measurement model was confirmed, composite scores for each variable were calculated and used in the analyses. Third, the PROCESS macro (Model 86) in SPSS 25.0 was used to examine the proposed mediation and moderated mediation effects. This approach is commonly used in organizational behavior studies, where validated measurement scales are summed to create composite scores that are then used in regression-based mediation and moderation analyses.

To test potential CMB, this study introduced a latent method factor into the measurement model. The results indicate that the inclusion of this method factor neither substantially improved the model fit nor significantly altered the standardized loadings of the substantive constructs, suggesting that CMB does not pose a substantial threat to the findings of this study.

## 4 Results

### 4.1 Descriptive statistics and correlation analysis

Table 2 presents the descriptive statistics and correlation values for all major variables, including means, standard deviations, and intercorrelations. The analysis revealed that JP exhibited significant positive correlations with PI (*r* = 0.741, *p* < 0.001), SE (*r* = 0.723, *p* < 0.001), and LMX (*r* = 0.596, *p* < 0.001). In addition, PI demonstrated strong positive associations with both SE (*r* = 0.736, *p* < 0.001) and LMX (*r* = 0.665, *p* < 0.001). Similarly, the correlation between SE and LMX was both significant and positive (*r* = 0.682, *p* = 0.01). These results offer preliminary empirical support for the proposed hypotheses.

**Table 2:** Descriptive statistical analysis and correlation of the four observed variables (*N* = 2,392).

Variable	M	SD	JP	PI	SE	LME
JP	3.9601	0.7504	1			
PI	4.0453	0.750	0.741^**^	1		
SE	3.81	0.757	0.723^**^	0.736^**^	1	
LME	3.706	0.842	0.596^**^	0.665^**^	0.682^**^	1

^*^p < 0.05,

^**^p < 0.01,

^***^p < 0.001.

### 4.2 Model testing and path analysis

#### 4.2.1 The mediating function of LMX and SE

Drawing upon the preliminary results in Table 3, PI and JP were respectively specified as the independent variable and dependent variable. LMX and SE were introduced as mediating variables, while age, marital status, academic position, educational attainment, and annual income were included as control variables.

**Table 3:** Results of main and mediation effect analyses.

Independent variables	Dependent variables								
	LMX			SE			JP		
	β	SE	*t*	β	SE	*t*	β	SE	*t*
Control variables									
Gender	−0.164	0.028	−5.9143^***^	−0.035	0.021	−1.671	−0.038	0.020	−1.893
Age	−0.002	0.020	−0.121	0.004	0.015	0.283	0.032	0.015	2.1662^*^
Marital status	0.046	0.035	1.328	0.044	0.026	1.676	0.003	0.025	0.111
Academic position	0.001	0.017	0.048	0.008	0.013	0.645	0.003	0.013	0.216
Educational attainment	−0.099	0.029	−3.432	−0.008	0.022	−0.382	−0.010	0.021	−0.471
Annual income	0.025	0.023	1.097	−0.033	0.017	−1.909	0.022	0.017	1.316
Predictor variables									
PI	0.745	0.018	42.1686^***^	0.527	0.018	30.067^6***^	0.480	0.020	24.158^***^
LMX				0.306	0.015	19.922^***^	0.046	0.016	2.885^*^
SE							0.335	0.020	16.965^***^
	R20 = 0.4445			R20 = 0.612			R20 = 0.634		
	F = 272.5732^***^			F = 469.998^***^			F = 458.083^***^		

^*^p < 0.05,

^**^p < 0.01,

^***^p < 0.001.

Regression analysis yielded a robust model fit (*R*^2^ = 0.574, *F* (7, 2,384) = 42.169, *p* < 0.001), with PI emerging as a significant and positive predictor of JP (β = 0.767, *t* = 55.627, *p* < 0.001), thereby providing empirical support for Hypothesis 1, which posits a strong positive link between PI and JP among VET teachers.

Subsequent path analysis (Table 3) further substantiated that PI exerted a significant positive effect on LMX (β = 0.745, *t* =42.169, *p* < 0.001), which, in turn, significantly predicted JP (β = 0.046, *t* = 2.885, *p* < 0.01), thereby indicating that LMX functions as a mediator in the relationship between PI and JP. Additionally, PI significantly predicted SE (β = 0.527, *t* = 30.068, *p* < 0.001), while LMX also exerted a positive effect on SE (β = 0.306, *t* = 19.922, *p* < 0.001); SE, in turn, subsequently predicted JP (β = 0.335, *t* = 16.965, *p* < 0.001), thereby indicating that SE also mediates the effect of LMX on JP.

These findings collectively provide empirical support for the chain mediation effect, whereby LMX and SE jointly transmit the effect of PI on JP among VET teachers.

According to the results displayed in Table 4, Indirect Effect 2 accounts for a larger proportion of the total effect than Indirect Effect 1. This pattern suggests that SE exerts a stronger mediating effect than LMX in the relationship between PI and JP among VET teachers. This finding further implies that, in the professional context of VET teachers, SE may constitute a more critical factor than LMX in influencing JP.

**Table 4:** Results of total, direct, and mediating effect analyses.

Path		Effect value	Proportion	BootSE	BootLLCI	BootULCI
Direct effect	PI → JP	0.480	0.625	0.020	0.441	0.519
Total indirect effect		0.288	0.375	0.022	0.245	0.331
Indirect effect 1	PI → LMX → JP	0.034	0.044	0.016	0.004	0.067
Indirect effect 2	PI → SE → JP	0.177	0.231	0.016	0.147	0.208
Indirect effect 3	PI → LMX → SE → JP	0.077	0.100	0.008	0.061	0.093

#### 4.2.2 Test of moderated chain mediation effect

Drawing upon the analytical framework presented in Table 5, this study employed the PROCESS macro (Model 86) for SPSS to examine the boundary conditional effects of JC in the relationship between PI and JP. The results revealed a significant interaction effect of PI and JC on LMX (β = 0.051, *p* < 0.001). Moreover, both PI (β = 0.428, *p* < 0.001) and JC (β = 0.436, *p* < 0.001) exerted significant main effects on LMX.

**Table 5:** Results of moderated chain mediation effect analyses.

Independent variables	Dependent variables					
	LMX			JP		
	β	SE	*t*	β	SE	*t*
Gender	−0.140	0.027	−5.2811^***^	−0.037	0.020	−1.891
Age	−0.008	0.019	−0.416	0.027	0.014	1.905
Marital status	0.029	0.033	0.856	−0.008	0.025	−0.316
Professional title	0.008	0.017	0.467	0.010	0.012	0.796
Educational background	−0.081	0.028	−2.922	−0.005	0.020	−0.258
Annual income	0.024	0.022	1.0788^*^	0.023	0.016	1.386
PI	0.428	0.030	14.180^***^	0.334	0.024	14.2121^***^
LMX	„	„	„	0.029	0.016	1.857
SE	„	„	„	0.231	0.022	10.7015^***^
JC	0.436	0.029	14.816^***^	0.265	0.025	10.4742^***^
Interaction terms	0.051	0.013	3.786^***^	−0.026	0.010	−2.5798^**^
	R20 = 0.492			R20 = 0.652		
	F = 256.510^***^			F = 405.610^***^		

^*^p < 0.05,

^**^p < 0.01,

^***^p < 0.001.

With respect to JP, the interaction between PI and JC was also significant (β = −0.026, *p* < 0.01), providing partial support for the hypothesized moderation. These results indicate that JC moderates specific components of the mediation, particularly the PI → LMX pathway, rather than exerting a consistent influence across all paths.

As presented in Table 6, this study further employed a bootstrapping procedure using the PROCESS to examine the role of JC in the chain mediation model. Regarding the simple mediation pathway (PI → LMX → JP), the indirect effects failed to reach statistical significance at either low [*effect* = 0.011, 95% *CI* (−0.004, 0.028)] or high levels of JC [*effect* = 0.014, 95% *CI* (−0.005, 0.034)], indicating that JC does not significantly moderate the simple mediation pathway.

**Table 6:** Results of moderated chain mediation effect analyses.

Path	JC level	Effect	BootSE	95% CI
PI → LMX → JP	Low	0.011	0.008	[−0.004, 0.028]
PI → LMX → JP	High	0.014	0.01	[−0.005, 0.034]
PI → LMX → SE → JP	Low	0.028	0.004	[0.020, 0.037]
PI → LMX → SE → JP	High	0.033	0.005	[0.023, 0.044]

In contrast, the sequential mediation pathway (PI → LMX → SE → JP), the indirect effects demonstrated robust significance at both low [*effect* = 0.028, 95% *CI* (0.020, 0.037)] and high levels of JC [*effect* = 0.033, 95% *CI* (0.023, 0.044)]. These results indicate that the sequential mediation effect remains stable and significant across varying levels of JC, with only minor variation in magnitude.

Notably, while the direct effect of LMX on JP failed to reach statistical significance, the sequential indirect effect remained significant. This underscores LMX's pivotal role as a relational resource capable of catalyzing the activation of SE, thereby promoting JP.

Although conditional indirect effects fluctuated alongside varying levels of JC, such descriptive variations fail to establish a statistically rigorous dependence on the moderator. Therefore, the index of moderated mediation was subsequently evaluated.

As detailed in Table 7, the moderated mediation index for the simple pathway (PI → LMX → JP) fell short of significance (*index* = 0.002, *Boot SE* = 0.001, 95% *CI* [−0.001, 0.004]). Conversely, the corresponding index for the sequential pathway (PI → LMX → SE → JP) proved robustly significant [*index* = 0.004, *Boot SE* = 0.001, 95% *CI* (0.002, 0.006)]. These findings indicate that JC exclusively moderates the sequential indirect pathway, with no empirical support for moderation in the simple pathway.

**Table 7:** Index of moderated mediation.

Indirect path	Index	BootSE	BootLLCI	BootULCI	Result
PI → LMX → JP	0.002	0.001	−0.001	0.004	Not significant
PI → LMX → SE → JP	0.004	0.001	0.002	0.006	Significant

**Figure 2:**
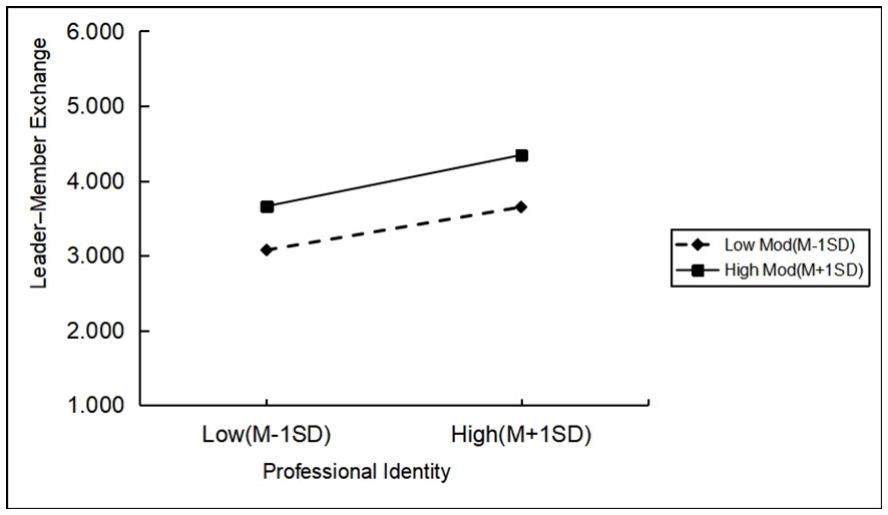
Interaction effect of professional identity and job crafting on leader–member exchange.

**Figure 3:**
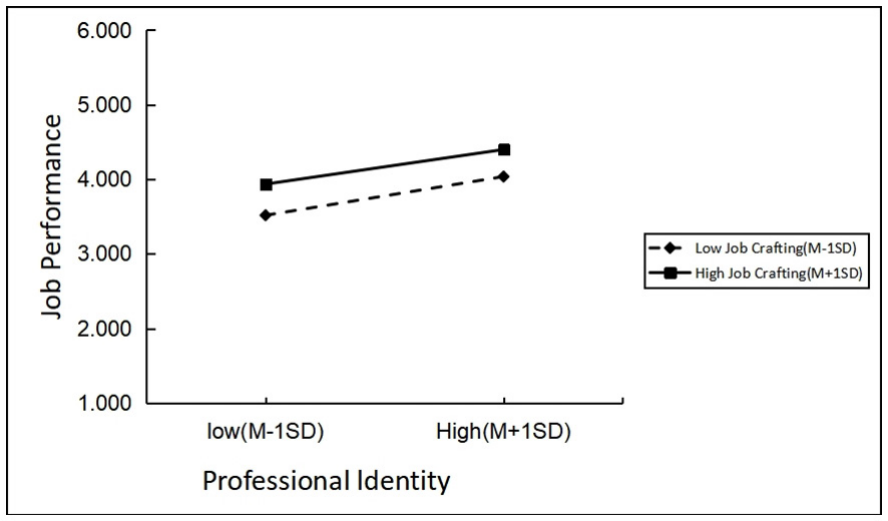
Interaction effect of professional identity and job crafting on job performance.

The moderating role of JC was also tested using simple slope analyses, as shown in Figures 2, 3. As illustrated in Figure 2, the positive effect of PI on LMX is stronger at higher levels of JC. Similarly, Figure 3 shows that JC also moderates the relationship between PI and JP in a similar fashion. But the size of these moderating effects is small. This could be due to the use of a more targeted measure of PI, and the inclusion of new data, which generally result in more conservative but more accurate estimates of interaction effects. As such, the moderating effect of JC should be viewed with caution due to its small practical significance.

In order to test the stability of the model, we also performed supplementary analyses by removing control variables that were not significantly associated, namely marital status, education and annual income. The results of this analysis were largely identical to the original model, with no meaningful differences in the estimates of the key variables. These findings therefore offer further evidence of the stability of the study findings.

## 5 Discussion

### 5.1 Discussion of results

Beyond validating the individual hypotheses, the findings elucidate the complete mechanism through which PI influences JP *via* systemic resource transfer. Specifically, PI functions through a dual mechanism involving relational (LMX) and psychological (SE) resources, thereby linking identity to behavioral outcomes across levels. More importantly, this process is further moderated by JC, which modulates the strength of the mediating pathways by influencing resource acquisition and transformation. Ultimately, these results suggest that teacher performance is not merely a direct consequence of PI, but rather emerges through the mobilization and transformation of various work-related resources.

#### 5.1.1 Direct effect of PI on JP

The findings unequivocally demonstrate that PI exerts a substantial influence on the JP of VET teachers, thereby providing robust support for Hypothesis 1. This finding corroborates the assertions of previous studies (Levin et al., 2022). Consistent with previous work, PI functions as a vital psychological resource that bolsters job satisfaction and facilitates individual's sense of professional mission (Wu et al., 2024). Teachers with a strong PI exhibit heightened commitment to high-quality teaching and research work; motivated by intrinsic dedication to teaching and their organizational attachments, hence achieving superior JP (Li et al., 2025). Furthermore, profound identification with one's organizational role enhances role clarity while reducing ambiguity and conflict (Ashforth and Mael, 1989). Such clarity empowers teachers to understand what they are expected to do, internalize the common purposes and shared goals among colleagues, and translate these shared objectives into an intrinsic motivation to excel, ultimately resulting in higher JP (De Backer et al., 2023).

#### 5.1.2 The mediating mechanism of LMX

Our results delineate LMX as a supplementary conduit linking PI to JP, and thereby partially supporting Hypothesis 2. Although the indirect effect channeled through LMX was statistically significant, its strength was demonstrably subordinate to that of SE's. Such a pattern suggests that LMX operates as a complementary, rather than a primary mechanism explaining the PI– JP nexus. Conceptually, individuals who identify strongly with their roles will be able to internalize organizational mission and vision, which in turn fosters deeper work engagement (Jiatong et al., 2022). This heightened engagement leads to more frequent and constructive interactions between employees and their leaders (Henderson et al., 2009). Fundamentally, high-quality LMX is characterized by positive, respectful, and supportive interactions with leaders. LMX relations among employees and their leaders lead to increased job satisfaction and organizational commitment, and, ultimately optimizing overall work performance (Willie, 2025).

#### 5.1.3 The mediating mechanism of SE

The findings support the notion that SE is a key mediator in the link between PI and JP, thus supporting Hypothesis 3. SE explains a much greater share of the indirect effect and is positioned more centrally in the model than LMX. In addition, the results reveal that LMX does not have a significant direct effect on JP, implying that its effects are largely indirect. This finding is in line with previous studies that highlight SE as a key psychological resource that connects PI to performance (Kazmi and Javaid, 2022).

#### 5.1.4 The sequential mediating role of LMX to SE

This study confirmed a substantial sequential mediation effect of LMX and SE on the impact of PI on JP, thus supporting Hypothesis 4. This result reveals a more intricate, multi-level mediation effect that has been overlooked in previous studies, thereby contributing to theory. For example, while prior research has shown the beneficial effect of high-quality LMX on PI (Fang et al., 2025), the current findings build on this research by confirming a sequential mediation effect of LMX and SE. More significantly, the results show that the indirect effect of SE is much stronger than that of LMX, suggesting its predominant role in the mediation process. In vocational education, teachers‘ job demands mainly focus on teaching. As a result, vocational education teachers are more likely to draw on their personal abilities to meet these job requirements (Silina-Jasjukeviča et al., 2025). Consequently, SE emerges as a critical determinant of JP. Taken together, our findings indicate that while LMX helps to construct the relational context for teachers' performance, SE plays a more pronounced role in relaying the influence of PI on JP.

#### 5.1.5 Moderating role of JC

The empirical evidence indicated that JC serves as a boundary condition influencing selected relationships within the model. Specifically, while the moderated mediation effect was not supported for the simple pathway (PI → LMX → JP), it was significant for the sequential pathway (PI → LMX → SE → JP), thereby supporting Hypotheses 5 and 6. These findings suggest that JC's moderating role is relatively limited in scope and primarily operates within the sequential indirect pathway rather than across all model pathways.

Admittedly, sparse empirical literature has directly suggested such a moderated chain mediation model. Nevertheless, the direction and statistical significance of the observed effects are consistent with prior findings in the literature. For instance, Wrzesniewski and Dutton (2001) posited that elevated levels of JC will fortify identity construction. Bhopal and Devi (2025) found that JC enhances the person–organization fit, work experience, and job satisfaction, while increasing SE. Complementarily, Moreira et al. (2022) claimed that JC helps individuals derive heightened competence and meaningfulness of work, which subsequently facilitates engagement and drives performance. Intriguingly, the data revealed that the interactive effect of PI and JC exerted divergent directional influences across different outcomes. Specifically, JC amplified the effect of PI on LMX, but attenuated its direct impact on JP.

These findings can be explained by COR Theory (Bai et al., 2021). When individuals engage in JC, they often invest more resources to acquire supplementary work-related resources (e.g., LMX and SE), thereby strengthens the indirect pathway. However, this process may also attenuate the direct effect of PI on JP. Overall, JC is more likely to reconfigure the mechanisms linking PI to JP rather than uniformly strengthening all pathways. Furthermore, while the moderating effect of JC is statistically significant, its magnitude remains modest and requires cautious interpretation.

### 5.2 Implications of the study

#### 5.2.1 Theoretical significance

First, the present study advances the existing literature by suggesting that PI not only exerts a direct influence on JP but also indirectly shapes it through mediating variables including LMX and SE. Consequently, this paper provides a more comprehensive empirical framework elucidating how PI drives JP among vocational education instructors. More importantly, this study reconceptualizes the PI-JP relationship as a structural process of resource flow and delineates the distinct roles of relational and psychological resources within this relationship.

Second, the findings delineate that LMX and SE influence teacher JP *via* divergent operational mechanisms. Specifically, LMX acts a crucial relational conduit mediating the influence of PI on teachers' JP. However, SE plays a more significant role in this context. This study further enriches the existing body of research.

Third, by integrating the moderating role of JC into the sequential mediation process, this study broadens the scope of existing research. The results thus indicate that JC functions as a boundary condition affecting selected relationships within the model, with its moderating effect primarily concentrated within the sequential pathway (PI → LMX → SE → JP), rather than being uniformly distributed across all the paths. Consequently, this finding provides a more nuanced understanding of the relationship between PI and JP.

#### 5.2.2 Practical insights

First, vocational education institutions should conceptualize teachers' PI as a fundamental resource for development; however, greater emphasis should be placed on leveraging PI to cultivate psychological resources, particularly SE in task performance. Accordingly, these institutions must move beyond conventional staff training by designing targeted interventions, such as on-the-job experiences, recognition mechanisms, and competency-based learning programs, to enhance teachers' task-specific confidence, given that self-confidence acts as the most potent driver of JP.

Second, while fostering LMX remains valuable, it should be conceptualized as a supportive mechanism to facilitate the development of teachers' internal resources. Administrators at vocational schools should cultivate environments and opportunities that empower teachers to seek feedback, gain recognition, and access professional development, thereby indirectly enhancing SE and improving JP.

Third, because JC selectively reinforces specific pathways rather than uniformly enhancing all mechanisms, interventions involving JC must be applied more selectively. Vocational schools should encourage teachers to prioritize in JC behaviors oriented toward resource acquisition (e.g., forging LMX and enhancing SE), but it is also important to recognize that excessive resource investment may not yield commensurate improvements in performance. Therefore, the implementation of effective and context-appropriate job designs is imperative.

## 6 Limitations and future directions

First, this study uses a cross-sectional design, and the analysis is mainly based on the relationships identified by factor analysis and path modeling. This type of design does not allow for strong causal claims. As such, future studies should consider longitudinal designs to include a time element and offer stronger grounds for causal inference.

Second, the link between PI and JP may be dynamic and bidirectional. This study investigates the impact of PI on JP; however, due to data limitations, the reciprocal relationship, that is, the impact of JP on PI, was not investigated. This relationship should be explicitly examined in future research to better understand their interaction.

Third, while several procedural and statistical techniques were applied to control for CMB, the sole use of single-source, self-reported data could still lead to bias. Although Harman's single-factor test and latent variable approaches suggested no significant common method variance, future studies could address this issue by using multi-source data or longitudinal data collection methods.

Fourth, while a number of demographic variables were controlled for, the choice of variables was somewhat arbitrary and not always well justified. Future research should take a more theoretically informed approach to the selection of control variables, ensuring that each variable is conceptually related to both the predictor and outcome variables, thus improving the validity and interpretability of the results.

Last, while the moderating effect of JC was significant, it was weak. As shown in the marginal slopes of the interaction plots, the moderating effect of JC may therefore be weak. Future studies should explore the moderating effects of JC under different circumstances, and more complex interaction effects.

## 7 Conclusion

In this study, a moderated serial mediation model was proposed to explore the underlying process of PI on the JP of vocational college teachers. The empirical findings revealed three main results.

First, PI was found to have a significant influence on JP. Second, a serial indirect pathway was found from PI to JP *via* LMX and SE, with SE serving as the stronger mediator in the indirect pathway. Third, JC was identified as a moderator of certain relationships in the model. Specifically, the moderating effect was mainly observed in the serial indirect pathway (PI → LMX → SE → JP), but not in all pathways. Furthermore, the size of the moderating effect was small, implying that its practical implications should be viewed with caution.

## Data Availability

The raw data supporting the conclusions of this article will be made available by the authors, without undue reservation.
