# Supplementary material for: Professional identity and job performance among vocational education teachers—a chain mediation model of leader–member exchange and self-efficacy, with job crafting as a moderator
Source: Front Psychol. 2026 Jun 5;17:1758017. doi: 10.3389/fpsyg.2026.1758017 (PMC13279083; doi:10.3389/fpsyg.2026.1758017)
Supplement: Supplementary file 1 [file Supplementary_file_1.docx]

## Questionnaire on the Current Work Status of Vocational Education Teachers

Dear Teacher,

Hello! Thank you very much for taking the time to participate in this survey. This study aims to explore vocational education teachers’ professional identity and their current work conditions. The data collected will be used solely for academic research and educational improvement purposes and will not involve any personal privacy.

This questionnaire is anonymous, and there are no right or wrong answers. Please respond based on your true feelings and experiences.

Thank you sincerely for your cooperation and support!

**Part II: Teacher Job Performance**

(Please select the number that best reflects your attitude and current situation.)

| questionnaire items | Scale:1 = Strongly Disagree  Scale:5 = Strongly Agree | | | | |
| --- | --- | --- | --- | --- | --- |
| I can accurately achieve my predetermined work goals | 1 | 2 | 3 | 4 | 5 |
| I can effectively utilize various resources at work | 1 | 2 | 3 | 4 | 5 |
| I work efficiently | 1 | 2 | 3 | 4 | 5 |
| I effectively implement departmental decisions | 1 | 2 | 3 | 4 | 5 |
| I can accurately complete tasks assigned by my supervisor | 1 | 2 | 3 | 4 | 5 |
| I handle various work tasks well | 1 | 2 | 3 | 4 | 5 |
| I can adapt well to different work environments and norms | 1 | 2 | 3 | 4 | 5 |
| I can creatively solve complex and novel problems at work | 1 | 2 | 3 | 4 | 5 |
| I continuously update my knowledge and skills to meet work demands | 1 | 2 | 3 | 4 | 5 |
| I can effectively deal with emergencies at work | 1 | 2 | 3 | 4 | 5 |
| I maintain an open and flexible attitude toward interpersonal interactions | 1 | 2 | 3 | 4 | 5 |

**Part III: Teacher Professional Identity**

(Please select the number that best reflects your attitude and current situation.)

| questionnaire items | Scale:1 = Strongly Disagree  Scale:5 = Strongly Agree | | | | |
| --- | --- | --- | --- | --- | --- |
| I believe that vocational education teaching is a highly professional occupation requiring systematic training | 1 | 2 | 3 | 4 | 5 |
| I actively follow information and public opinions about vocational education teachers | 1 | 2 | 3 | 4 | 5 |
| I believe teachers’ professional competence greatly influences student development | 1 | 2 | 3 | 4 | 5 |
| I care about how others perceive vocational education teachers | 1 | 2 | 3 | 4 | 5 |
| I believe there is no essential difference between vocational and higher education teachers | 1 | 2 | 3 | 4 | 5 |
| I believe I am suitable for the teaching profession and can become an excellent vocational teacher | 1 | 2 | 3 | 4 | 5 |
| My subject knowledge meets teaching requirements | 1 | 2 | 3 | 4 | 5 |
| I possess pedagogical and psychological knowledge applicable to teaching | 1 | 2 | 3 | 4 | 5 |
| I have practical experience and skills required for teaching | 1 | 2 | 3 | 4 | 5 |
| I can reflect and conduct inquiry to improve teaching | 1 | 2 | 3 | 4 | 5 |
| I am capable of continuous learning and professional development | 1 | 2 | 3 | 4 | 5 |
| I actively learn teaching methods and classroom management from others | 1 | 2 | 3 | 4 | 5 |
| I take school-arranged teaching, research, and training activities seriously | 1 | 2 | 3 | 4 | 5 |
| I actively seek opportunities (e.g., open classes, competitions) to improve my teaching | 1 | 2 | 3 | 4 | 5 |
| I continuously enrich my knowledge through reading and learning | 1 | 2 | 3 | 4 | 5 |
| I have clear and specific professional development goals | 1 | 2 | 3 | 4 | 5 |
| I am willing to remain in the teaching profession long-term | 1 | 2 | 3 | 4 | 5 |
| Teaching makes me feel energetic and accomplished | 1 | 2 | 3 | 4 | 5 |
| Working in vocational education is meaningful and valuable to me | 1 | 2 | 3 | 4 | 5 |
| I believe becoming a teacher is a correct career choice | 1 | 2 | 3 | 4 | 5 |
| Being a teacher gives me social recognition and status | 1 | 2 | 3 | 4 | 5 |
| I believe caring for students and promoting their holistic development is the core value of teaching | 1 | 2 | 3 | 4 | 5 |

**Part IV: Job Crafting**

(Please select the number that best reflects your attitude and current situation.)

| questionnaire items | Scale:1 = Strongly Disagree  Scale:5 = Strongly Agree | | | | |
| --- | --- | --- | --- | --- | --- |
| I voluntarily take on additional tasks at work | 1 | 2 | 3 | 4 | 5 |
| I add tasks that match my interests or skills | 1 | 2 | 3 | 4 | 5 |
| I prioritize tasks aligned with my interests or skills | 1 | 2 | 3 | 4 | 5 |
| I proactively modify the scope or nature of my tasks | 1 | 2 | 3 | 4 | 5 |
| I introduce new methods to improve my work | 1 | 2 | 3 | 4 | 5 |
| I try to better understand people at work (colleagues and supervisors) | 1 | 2 | 3 | 4 | 5 |
| I build relationships with colleagues who share similar interests/skills | 1 | 2 | 3 | 4 | 5 |
| I participate in work-related social activities | 1 | 2 | 3 | 4 | 5 |
| I mentor and support new colleagues | 1 | 2 | 3 | 4 | 5 |
| I reflect on the meaning of my work in life | 1 | 2 | 3 | 4 | 5 |
| I remind myself of the importance of my work to the school | 1 | 2 | 3 | 4 | 5 |
| I remind myself of the importance of my work to students and others | 1 | 2 | 3 | 4 | 5 |
| I reflect on how my work positively influences my life | 1 | 2 | 3 | 4 | 5 |
| I reflect on how my work affects my well-being | 1 | 2 | 3 | 4 | 5 |

**Part V: Leader–Member Exchange**

(Please select the number that best reflects your attitude and current situation.)

| 项目 | Scale:1 = Strongly Disagree  Scale:5 = Strongly Agree | | | | |
| --- | --- | --- | --- | --- | --- |
| My supervisor understands my potential | 1 | 2 | 3 | 4 | 5 |
| I can clearly express my position when interacting with my supervisor | 1 | 2 | 3 | 4 | 5 |
| My supervisor understands my work-related needs and problems | 1 | 2 | 3 | 4 | 5 |
| My supervisor uses their authority to help me solve major work problems | 1 | 2 | 3 | 4 | 5 |
| I can rely on my supervisor to support me when I truly need help | 1 | 2 | 3 | 4 | 5 |
| My supervisor has confidence in me and would defend my decisions | 1 | 2 | 3 | 4 | 5 |

End of Questionnaire – Thank you for your participation!
